# Elucidating the Role of NaCl in the on‐Surface Synthesis of Conjugated Azaacene Polymers on Au(111)

**DOI:** 10.1002/chem.202502452

**Published:** 2025-11-05

**Authors:** Tim Kratky, Xunshan Liu, Sebastian Günther, Pingo Mutombo, Luca Schio, Luca Floreano, Silvio Decurtins, Jascha Repp, Pavel Jelínek, Shi‐Xia Liu, Laerte L. Patera

**Affiliations:** ^1^ Department of Chemistry and Catalysis Research Center Technical University of Munich 85748 Garching Germany; ^2^ Department of Chemistry, Biochemistry and Pharmaceutical Sciences, W. Inäbnit Laboratory for Molecular Quantum Materials and Werner Siemens Center for Molecular Quantum Systems University of Bern Bern 3012 Switzerland; ^3^ Department of Chemistry Zhejiang Sci‐Tech University Hangzhou 310018 China; ^4^ Institute of Physics of Czech Academy of Sciences Prague 16200 Czech Republic; ^5^ CNR – Istituto Officina dei Materiali (IOM) TASC Laboratory Trieste 34149 Italy; ^6^ Institute of Experimental and Applied Physics University of Regensburg 93053 Regensburg Germany; ^7^ Regional Centre of Advanced Technologies and Materials, Czech Advanced Technology and Research Institute (CATRIN) Palacký University Olomouc Olomouc 78371 Czech Republic; ^8^ Department of Physical Chemistry University of Innsbruck Innsbruck 6020 Austria

**Keywords:** density functional theory, on‐surface synthesis, scanning tunneling microscopy, surface chemistry, X‐ray photoemission spectroscopy

## Abstract

On‐surface synthesis (OSS) offers unique opportunities for fabricating carbon‐based nanostructures that are unattainable by conventional wet‐chemical synthesis. Despite OSS being extremely successful, the use of coadsorbates to promote reactions remains largely unexplored. In this study, we investigate the role of sodium chloride (NaCl) in promoting the Scholl reaction (oxidative aryl–aryl coupling) of hexaazatriphenylene (HAT) molecules on Au(111), leading to the growth of conjugated azaacene oligomers. Using scanning tunneling microscopy (STM), synchrotron‐based X‐ray photoelectron spectroscopy (XPS), and density functional theory (DFT) calculations, we shed light on the reaction mechanism and the intermediates involved. Upon codeposition on Au(111), NaCl decomposes on the surface, releasing Na atoms that form thermally stable metal‐organic complexes, enhancing precursor stability against desorption. This stabilizing effect allows HAT molecules to undergo regioselective intermolecular coupling for polymerization at elevated temperatures. This study highlights the role of alkali metals in on‐surface chemical reactions and outlines a strategy for overcoming the precursor‐desorption issue.

## Graphical abstract

NaCl‐assisted on‐surface reaction on Au(111): NaCl decomposition yields Na adatoms that form metal–organic complexes, which stabilize the organic precursor, steering aryl–aryl coupling.

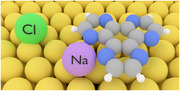

## 1 Introduction

On‐surface synthesis (OSS) of carbon‐based nanostructures has rapidly emerged as a powerful method for synthesizing nanomaterials with structures and functionalities that cannot be achieved by wet chemistry.^[^ ^1^, ^2^ ^]^ Ullmann coupling is one of the most commonly used chemical reactions, in which dehalogenation of suitably prefunctionalized precursors is exploited to create reactive intermediates for subsequent polymerization.^[^ ^3^ ^]^ Dehydrogenative homocoupling reactions have also been reported to yield polymeric networks.^[^ ^4^, ^5^, ^6^ ^]^ The limited size of precursors together with a high temperature required for the reaction can lead to desorption of the precursors, hampering the formation of extended nanostructures.^[^ ^7^ ^]^ This precursor‐desorption issue represents a significant challenge in OSS. The substrate plays often a crucial role in the entire process by mediating precursor activation, diffusion, and coupling.^[^ ^8^, ^9^ ^]^ Recent studies highlight also the importance of metal adatoms in unlocking previously inaccessible reaction pathways.^[^ ^10^, ^11^, ^12^, ^13^, ^14^ ^]^ Alkali metals, extensively employed as promoters in heterogeneous catalysis for enhancing activity and selectivity,^[^ ^15^, ^16^, ^17^ ^]^ were recently presented as a possible solution to the problem of precursor desorption.^[^ ^18^ ^]^ Metal‐organic nanostructures on atomically flat surfaces have been achieved by the complexation of alkali metals derived either from the deposition of their pure form^[^ ^19^, ^20^, ^21^, ^22^, ^23^ ^]^ or from alkali halides.^[^ ^24^, ^25^, ^26^, ^27^, ^28^, ^29^, ^30^, ^31^ ^]^ Specifically, alkali halides dissolve upon deposition,^[^ ^32^ ^]^ releasing alkali atoms that coordinate with molecular groups through electrostatic interactions.^[^ ^23^, ^24^ ^]^ Meanwhile, halogen atoms appear to remain on the surface, showing no involvement in interactions within the metal‐organic layer.^[^ ^24^ ^]^ While much effort has been devoted to control the formation of metal‐organic layers by complexation with alkali atoms^[^ ^24^, ^25^, ^26^, ^27^, ^28^, ^29^, ^30^, ^31^ ^]^ and more recently to metalation processes within porphyrins,^[^ ^33^ ^]^ little is known about their role in on‐surface coupling reactions.^[^ ^34^ ^]^ Recently, the codeposition of NaCl has been shown to promote regioselective homocoupling of hexaazatriphenylene (HAT) molecules on an Ag(111) surface.^[^ ^18^ ^]^ Complexation with Na atoms increases the HAT precursors’ thermal resistance to desorption, allowing them to reach temperatures at which the C─H bond is activated.^[^ ^18^ ^]^ However, despite the success of this approach, questions remained about the detailed reaction pathways. Density functional theory (DFT) calculations suggested the formation of stable (HAT)_2_‐Na complexes, however, direct experimental evidence for such intermediates has not been reported so far.

**Scheme 1:**
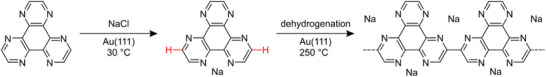
A schematic illustration of the Scholl reaction leading to the on‐surface oligomerization of HAT molecules on Au(111) in the presence of NaCl, whereby at least one N^N pocket for each HAT is coordinated with Na.

The primary aim of this work is to show that NaCl enhances the surface reactivity of HAT molecules by releasing Na adatoms that stabilize precursors and promote covalent coupling, thereby enabling Scholl‐type polymerization (oxidative aryl–aryl coupling) on the otherwise inert Au(111) surface (see Scheme 1). This reaction was specifically chosen because it exhibits the aforementioned precursor‐desorption issue and does not proceed on a bare Au(111) surface, making it an ideal platform for investigating the activity of alkali metals in OSS. *In*‐*situ* X‐ray photoelectron spectroscopy (XPS) was employed to monitor the elemental and chemical surface composition during the reaction, revealing the formation of metal‐organic complexes. The stability of these reaction intermediates was further detailed by DFT calculations, while high‐resolution scanning tunneling microscopy (STM) provided insights into the structures of the reaction products.

## 2 Results and Discussion

**Figure 1:**
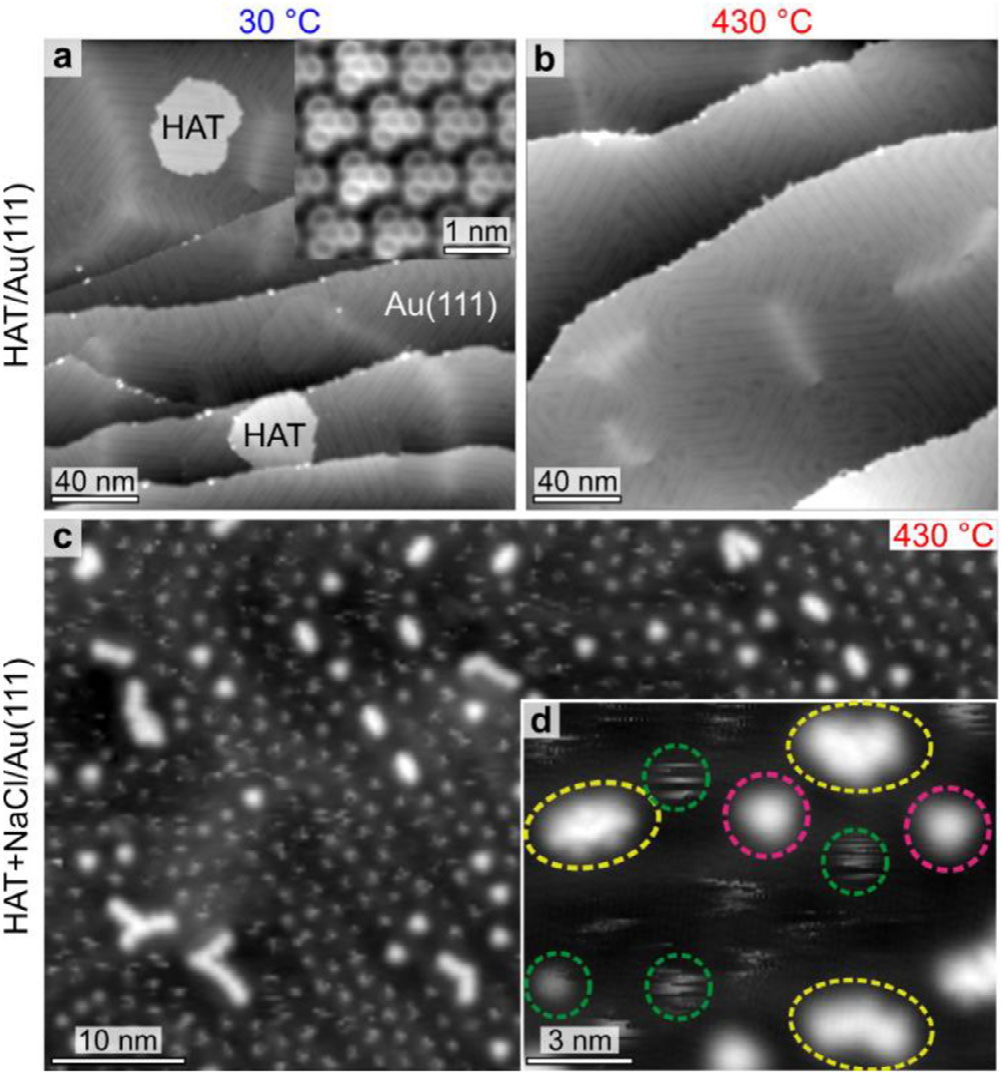
Effect of NaCl on the on‐surface synthesis of conjugated azaacene oligomers on Au(111). a, b) constant‐current STM images acquired after deposition of HAT molecules and annealing at 30 °C a) and 430 °C b). Inset in a): constant‐height AFM image of the HAT monolayer acquired with a CO‐functionalized tip. c,d) constant‐current STM images acquired upon deposition of submonolayer coverages of HAT and NaCl and annealing at 430 °C. d) oligomers, monomers, and atomic species are highlighted in yellow, pink and green, respectively. Measurement parameters: a) tunneling current *I *= 1.5 pA, sample bias voltage *V *= 1.0 V. Inset: z‐offset of 2.7 Å with respect to an STM set‐point of *I* = 1.5 pA, *V*  = 1 V. d) b) *I *= 1.7 pA, *V *= 1.0 V. c) *I *= 1.5 pA, *V *= 0.1 V. d) *I * =  1.1 pA, *V* =  1.0 V.

Figure 1a shows an STM image obtained upon deposition of a submonolayer of HAT molecules onto Au(111) and annealing at 30 °C, resulting in the formation of an extended crystalline assembly driven by hydrogen bonding. The inset presents a CO‐functionalized‐tip AFM image that directly resolves the intramolecular bonds within the HAT assembly.^[^ ^35^, ^36^ ^]^ The herringbone reconstruction remains visible below HAT assemblies, indicating a weak interaction of the monomers with the Au(111) surface. Annealing at 430 °C results in the complete desorption of the molecular layer (Figure 1b).^[^ ^18^ ^]^ In contrast, codepositing HAT precursors with NaCl and annealing at the same temperature yields a mixture of oligomers, monomers, and smaller species (Figure 1c, d). The latter, characterized by a very confined, diffuse, and fuzzy appearance, arrange into a locally hexagonal lattice without long‐range order (Figure 1c), consistent with mutual repulsive interactions indicative of individual charged adatoms. While these features strongly suggest the presence of charged species, precise identification from STM images alone remains challenging. Importantly, tip‐induced diffusion of these species does not prevent submolecular resolution of the monomer and dimer structures (see below).

**Figure 2:**
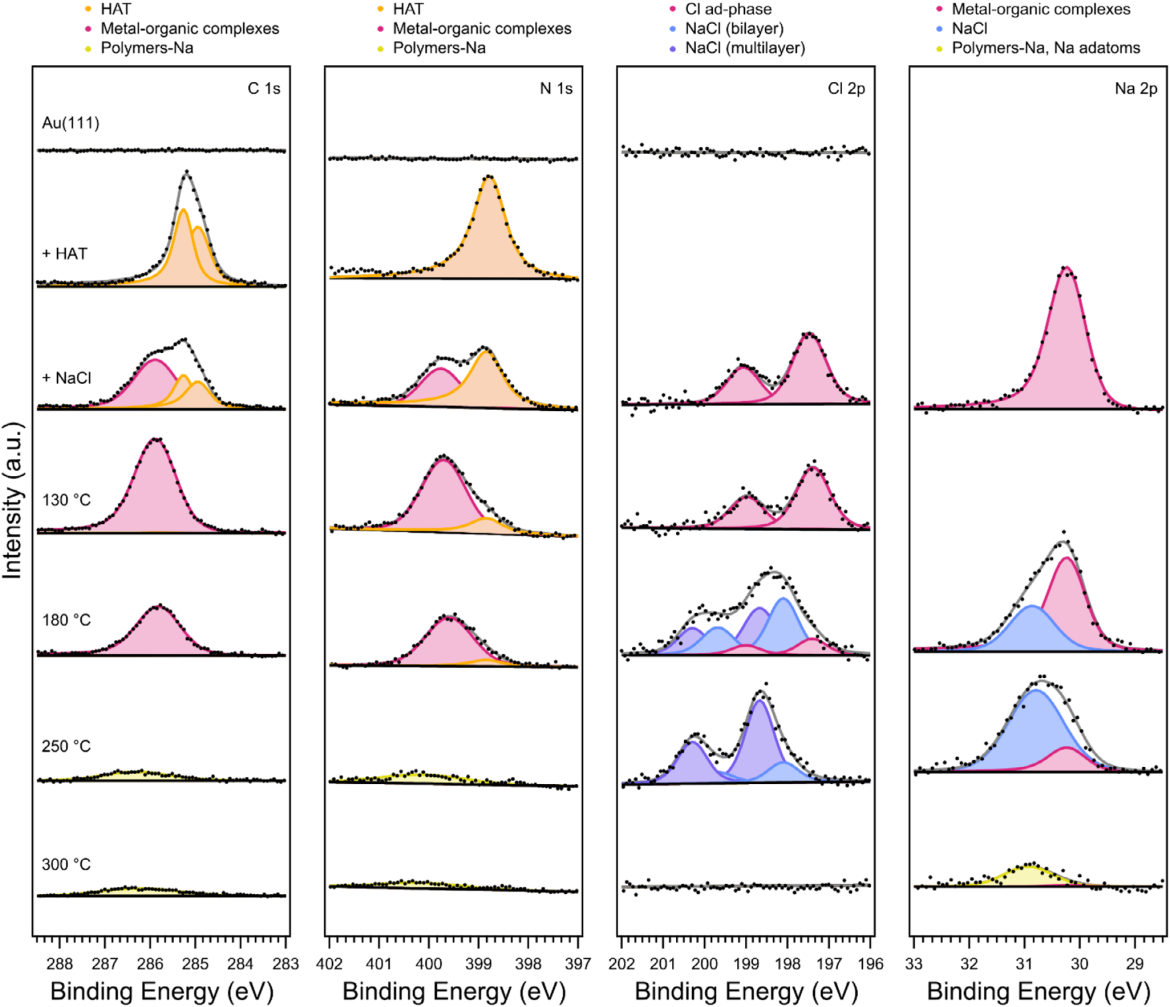
High‐resolution XP spectra of the C 1s, N 1s, Cl 2p, and Na 2p core levels acquired at room temperature upon deposition of HAT and NaCl and subsequent annealing at 130, 180, 250, and 300 °C for 1 minute each. Photon energies: 515 eV (energy resolution, Δ*E*  =  160 meV) for C 1s, N 1s, and Cl 2p, 200 eV (Δ*E* = 115 meV) for Na 2p. Spectral components are colored according to the corresponding surface species (see legend at the top), with the fitting results shown in light gray.

To elucidate the reaction pathway and chemically identify the reaction products, we performed synchrotron‐based XPS at the ALOISA beamline^[^ ^37^ ^]^ of the Elettra Synchrotron radiation facility (Trieste, Italy). Figure 2 shows XP spectra of the C 1s, N 1s, Cl 2p, and Na 2p core levels acquired at 30 °C upon deposition of HAT and NaCl at 30 °C and subsequent annealing at 130, 180, 250, and 300 °C for 1 minute each.

The C 1s spectrum of a HAT sub‐monolayer (0.5 monolayer, ML) on Au(111) comprises two main components of the same intensity, corresponding to the two chemically distinct carbon atoms in the central benzene and outer pyrazine rings of the HAT molecules. The N 1s spectrum exhibits only one feature at 398.8 eV, consistent with the high symmetry of HAT. Notably, upon deposition of NaCl (<0.1 bilayer, BL), additional, spectrally broad components at higher binding energies emerge in both the C 1s (285.8 eV) and N 1s (399.7 eV) regions, while the intensities of the HAT‐related components decrease. As the total photoemission intensities from the C 1s and the N 1s core levels remain constant, this suggests a conversion of HAT molecules into new species. Upon deposition of NaCl on HAT/Au(111), the Na 2p spectrum exhibits a single component at 30.2 eV, while the Cl 2p spectrum shows a single spin‐orbit doublet. The Cl 2p_3/2_ binding energy of 197.4 eV is lower than the values typically found for NaCl on Au(111) (>197.8 eV, see Figure S1), but aligns with the binding energy reported for atomic Cl adsorbed on Au(111).^[^ ^38^ ^]^ The emergence of new HAT‐related species in the C 1s and N 1s spectra suggests that NaCl decomposes on the surface,^[^ ^24^ ^]^ releasing Na atoms that interact with HAT molecules to form metal‐organic complexes. The broadening of the C 1s and N 1s peaks after NaCl co‑deposition arises likely from a continuous range of slightly different chemical environments experienced by HAT molecules upon Na complexation. Concurrently, Cl atoms contribute to the formation of a Cl/Au(111) ad‐phase.^[^ ^39^ ^]^ Interestingly, the components in the C 1s and N 1s core levels associated with HAT molecules on Au(111) disappear almost entirely upon annealing at 130 °C, indicating that the HAT molecules adsorbed on the Au surface are converted into a HAT‐Na structure. Since NaCl has already decomposed entirely during deposition at 30 °C, the subsequent formation of metal‐organic structures likely involves the incorporation of additional HAT molecules into the present metal‐organic complexes species (see below). At 180 °C, the overall C 1s and N 1s intensities decrease slightly without affecting the XPS line shapes, while new components in the Na 2p and Cl 2p core levels appear at higher binding energies. The Na 2p binding energy of 30.8 eV as well as the Cl 2p_3/2_ binding energies of the two new spin‐orbit doublets at 198.1 and 198.7 eV correspond to the formation of bilayer and multilayer NaCl films on Au(111), respectively (see Figure S1).^[^ ^40^, ^41^, ^42^ ^]^ Upon annealing at 250 °C, the C 1s and N 1s intensities decrease to about 15% of their initial values, accompanied by a binding energy shift to higher values (286.3 eV for C 1s and 400.1 eV for N 1s). In contrast, the Na 2p and Cl 2p intensities remain largely unchanged. The Na 2p signal associated with NaCl increases at the expense of the component linked to metal‐organic structures, while the Cl 2p signal from Cl ad‐phase disappears. Additionally, the intensity ratio of the two NaCl‐related components shifts toward the higher binding energy component, consistent with the thickening of the NaCl film observed on Au(111) above 160 °C (see Figure S1).^[^ ^42^ ^]^ No further significant loss of C 1s or N 1s signal intensity is observed upon annealing to 300 °C (Figure 2); rather, both peaks exhibit modest binding‐energy shifts and broadening consistent with onset of polymerization. At this stage, a small fraction of Na remains on the surface, while Cl signal falls below the detection limit.

The experimental observations reveal a complex reaction process involving competing mechanisms, which can be interpreted as follows. Upon the codeposition of HAT and NaCl, shifts in the binding energy of HAT‐related components suggest interactions between the N^N chelating pockets (i.e., the bidentate nitrogen coordination sites) of HAT molecules and Na atoms. These interactions result in the formation of metal‐organic complexes, leveraging up to three N^N chelating pockets present in the HAT molecules. Concurrently, adsorbed Cl forms a Cl/Au(111) ad‐phase.^[^ ^39^ ^]^ The absence of NaCl‐related components implies that NaCl decomposes entirely already during deposition at 30 °C. For the dataset shown in Figure 2, the amount of available Na compared to the number of N^N pockets of the HAT molecules limits the Na uptake into metal‐organic complexes. The complete disappearance of HAT‐related components after annealing at 180 °C is attributed to the formation of metal‐organic complexes which share a Na adatom, as (HAT)_2_‐(Na)_5_. The decrease in the C 1s and N 1s core level intensities at 180 °C suggests partial dissociation of the metal‐organic complexes, accompanied by the desorption of a fraction of the HAT molecules. At 180 °C, HAT desorption from Au(111) begins (see Figure S2), while, based on previous temperature‐programmed desorption studies,^[^ ^38^, ^43^ ^]^ Na and Cl are expected to remain surface, bound up to ∼ 430 °C and ∼ 370 °C, respectively, far above the maximum annealing temperature used here. Thus, the released Na atoms remain on the Au surface, recombining with the Cl ad‐phase to (re‐)form NaCl. At 250 °C, a further decrease in the C 1s and N 1s signal intensities indicates increased dissociation of complexes, resulting in additional HAT desorption and formation of NaCl on the Au(111) surface. The binding energy shifts in the C 1s and N 1s spectra provide clear evidence of the development of a new structure, likely HAT oligomers or polymers. This indicates that the desorption of HAT molecules competes directly with their polymerization at elevated temperatures.^[^ ^18^ ^]^ The NaCl layers remain stable up to 300 °C, where desorption sets in (see Figure S1). Remarkably, a small fraction of Na remains on the surface after annealing at 300 °C, while Cl has entirely vanished. This suggests that partial desorption of the Cl ad‐phase (both as Cl and Cl_2_ species)^[^ ^39^ ^]^ prevents all Na atoms, released by the decomposing metal‐organic complexes, from recombining with Cl to form NaCl and desorbing. Additionally, a fraction of Na likely remains bound to the polymer structures.^[^ ^18^ ^]^ We may conclude that polymerization of HAT molecules mediated by metal‐organic complexes takes place by conversion of sub‐ML coverages of HAT in the presence of a minor amount of NaCl on Au(111). The reaction of higher coverages of both HAT (> 1 ML) and NaCl (≈0.3 BL) was investigated using temperature‐programmed XPS experiments (see Supporting Information), indicating that the codeposited NaCl decomposes in a self‐limiting process delivering only the amount of Na required for the HAT complexation while the released Cl atoms populate an adsorbate phase.

**Figure 3:**
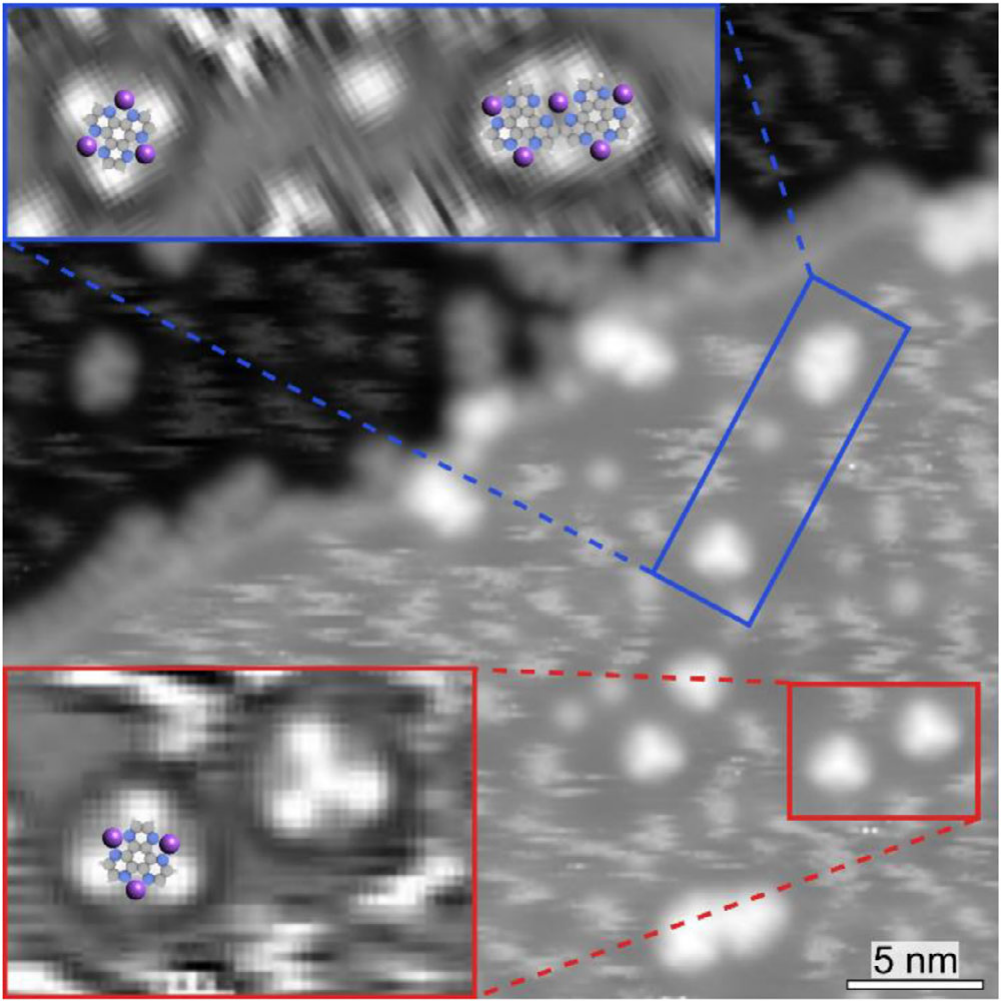
Metal‐organic complexes and oligomers. Constant‐current STM image acquired after deposition of HAT molecules and NaCl and annealing at 430 °C. Insets: Laplace filtered zoom‐in images. *I *= 1.1 pA, *V* =  1.0 V. Purple, grey, blue, and white spheres represent sodium, carbon, nitrogen, and hydrogen atoms, respectively.

The comparison of the XP spectra in Figure 2 with the STM images in Figure 1c, d allows for the identification of the reaction intermediate and products. While the atomic species consist of Na adatoms, the short chains correspond to oligomers of covalently bonded HAT precursors. The monomeric species, which exhibit threefold symmetric STM contrast (see Figure 3) are tentatively identified as HAT‐Na_3_ complexes which did not undergo polymerization. Although individual Na adatoms are not directly resolved, these structures clearly differ from the packing of pristine HAT molecules, indicating a distinct chemical identity. Given the challenges of visualizing adatoms by STM, our assignment of metal‐organic complex formation relies primarily on the C 1s and N 1s XPS signatures. Overlaying structural models on the Laplace‐filtered images reveals that the dimer (blue highlighted region in Figure 3) resembles the product previously reported upon dehydrogenation of pyrazino[2,3‐f][4,7]phenanthroline (pap) molecules on Au(111), where an aryl–aryl bond is formed.^[^ ^5^ ^]^

An important finding relates to the experimental observation of these monomeric species after annealing at a temperature well above the desorption temperature of HAT molecules (*T*  =  180 °C, see Figure S2). As previously reported by DFT calculation,^[^ ^18^ ^]^ the complexation of Na improves the thermal robustness to the desorption of adsorbed HAT species. This in turn creates likely conditions for favorable polymerization initiated by C─H dissociation. The resulting reactive species triggers intermolecular covalent coupling between HAT units, leading to the formation of the short oligomers, as the ones resolved in Figure 1d.

**Figure 4:**
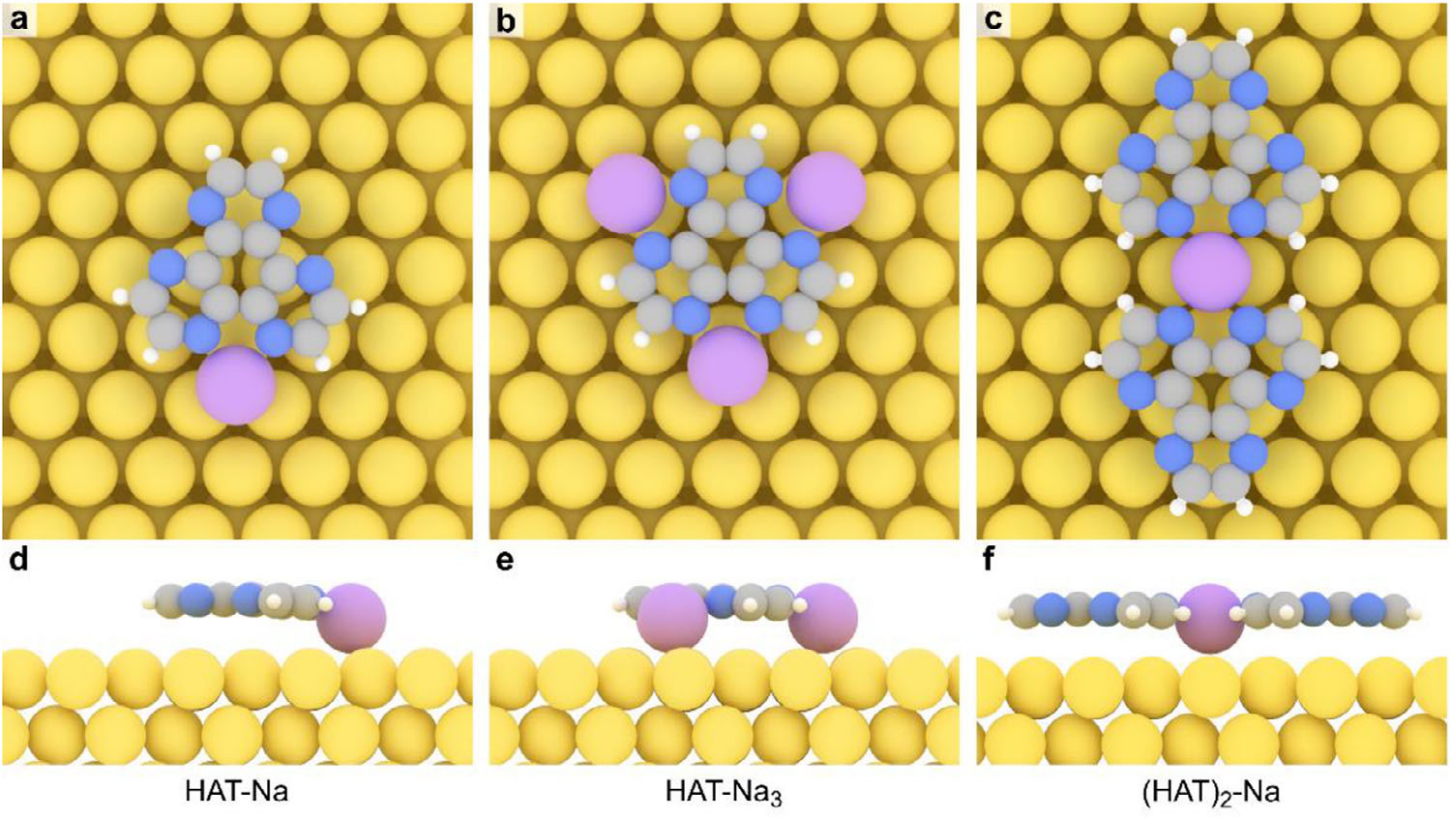
Optimized geometries of the HAT‐Na (left), HAT‐Na_3_ (middle) and (HAT)_2_‐Na (right) complexes. a,b,c) Top and d,e,f) side views. Purple, yellow, grey, blue, and white spheres represent sodium, gold, carbon, nitrogen, and hydrogen atoms, respectively.

To rationalize these observations, we turned to DFT calculations.^[^ ^44^ ^]^ First, we addressed the presence of charged Na species on the surface upon OSS. The calculated Hirshfeld charge of a Na adatom on Au(111) reveals the charge character (+0.39 e), as shown in Table S1. We then considered the energetics of complexes resulting from the coordination of HAT molecules with metal adatoms (Table S2). Complexation with a single Na adatom is predicted to be stable, with calculated binding energies of ‐2.87 eV for the HAT‐Na complex and ‐5.64 eV for the (HAT)_2_‐Na complex, while HAT‐Na_3_ exhibits a binding energy of ‐4.69 eV. The relaxed geometries of the three complexes are shown in Figure 4. The formation of bonding motifs with Au leads to less favorable configurations, similar to the case of Ag(111).^[^ ^18^ ^]^ Although pyridyl–Au adatom complexes have been previously reported,^[^ ^5^ ^]^ we observe no evidence for substantial Au─N coordination here: XPS indicates Na release (Figure 2) and DFT (Table S2) yields higher binding energies for Na at the chelating N sites than for Au, consistent with Na─N coordination being thermodynamically preferred. The high charge density of the complexes (Table S1) increases the binding with the Au substrate. The high stability and the threefold symmetry of the HAT‐Na_3_ complex (Figure 4b, e) strongly support its formation on the surface following NaCl dissolution (see monomeric species in Figure 3), with Na adatoms occupying each N^N chelating pocket of the HAT molecule.

While the formation of metal‐organic complexes is thermodynamically favored, kinetic barriers associated with the dissolution of the NaCl lattice must be overcome. Interestingly, it has been shown that NaCl bilayer islands on Au(111) begin to dissolve at temperatures as low as ‐130 °C when exposed to a water monolayer.^[^ ^32^ ^]^ Our XPS data (Figure 2) clearly reveals that NaCl dissolution mediated by HAT molecules is already taking place at 30 °C, highlighting the ability of HAT to facilitate this process under mild conditions.

## 3 Conclusion

In conclusion, we have elucidated the role of NaCl in tuning on‐surface reactivity by selectively stabilizing molecular precursors, thereby enabling Scholl‐type polymerization of HAT on the Au(111) surface, which is chemically inert toward such reaction. For HAT molecules on Au(111), the inherently low adsorption energies result in desorption at temperatures below those required for C─H bond activation, presenting a significant kinetic limitation for OSS, particularly on rather inert surfaces. Our study illustrates how Na complexation with precursor molecules effectively addresses the desorption issue^[^ ^7^ ^]^ by stabilizing the intermediates, allowing reactions to proceed. These findings underscore the potential of NaCl to overcome desorption barriers and promote the formation of conjugated nanostructures on inert surfaces, offering a versatile strategy for advancing on‐surface synthetic methodologies.

## Supporting Information

The authors have cited additional references within the Supporting Information.^[^ ^45^, ^54^ ^]^

## Conflict of Interest

The authors declare no conflict of interest.

## Supporting information



Supporting Information

## Data Availability

The data that support the findings of this study are openly available in [NAME] at [DOI], reference number [REF].
