# Supplementary material for: Elucidating the Role of NaCl in the on‐Surface Synthesis of Conjugated Azaacene Polymers on Au(111)
Source: Chemistry. 2025 Nov 5;31(69):e02452. doi: 10.1002/chem.202502452 (PMC12699165; doi:10.1002/chem.202502452)
Supplement: Supplementary file 1 — Supporting Information [file CHEM-31-e02452-s001.docx]

Supporting Information
©Wiley-VCH 2021
69451 Weinheim, Germany

Elucidating the role of NaCl in the on-surface synthesis of conjugated azaacene polymers on Au(111)

Tim Kratky,^[a]^ Xunshan Liu,^[b,c]^ Sebastian Günther,^[a]^ Pingo Mutombo,^[d]^ Luca Schio,^[e]^ Luca Floreano,^[e]^ Silvio Decurtins,^[b]^ Jascha Repp,^[f]^ Pavel Jelínek,*^[d,g]^ Shi-Xia Liu*^[b]^ and Laerte L. Patera*^[a,f,h]^

[a] T. Kratky, Prof. Dr S. Günther, Prof. Dr. L. L. Patera
Department of Chemistry and Catalysis Research Center
Technical University of Munich
85748 Garching (Germany)

[b] Prof. Dr. X. Liu, Prof. Dr. S. Decurtins, Dr. S.-X. Liu
Department of Chemistry, Biochemistry and Pharmaceutical Sciences
W. Inäbnit Laboratory for Molecular Quantum Materials and Werner Siemens Center for Molecular Quantum Systems
University of Bern
3012 Bern (Switzerland)
E-mail: [shi-xia.liu@unibe.ch](mailto:shi-xia.liu@unibe.ch)

[c] Prof. Dr. X. Liu
Department of Chemistry
Zhejiang Sci-Tech University
310018 Hangzhou (China)

[d] Dr. P. Mutombo, Prof. Dr. P. Jelinek
Institute of Physics of Czech Academy of Sciences
16200 Prague (Czech Republic)
E-mail: [jelinekp@fzu.cz](mailto:jelinekp@fzu.cz)

[e] Dr. L. Schio, Dr. L. Floreano
CNR - Istituto Officina dei Materiali (IOM)
TASC Laboratory
34149 Trieste (Italy)

[f] Prof. Dr. J. Repp, Prof. Dr. L. L. Patera
Institute of Experimental and Applied Physics
University of Regensburg
93053 Regensburg (Germany)

[g] Prof. Dr. P. Jelinek
Regional Centre of Advanced Technologies and Materials
Czech Advanced Technology and Research Institute (CATRIN)
Palacký University Olomouc
78371 Olomouc (Czech Republic)

[h] Prof. Dr. L. L. Patera
Department of Physical Chemistry
University of Innsbruck
6020 Innsbruck (Austria)
E-mail: [laerte.patera@uibk.ac.at](mailto:laerte.patera@uibk.ac.at)

**Experimental Procedures**

**Chemicals.** Dipyrazino[2,3-f][2’,3’-h]quinoxaline (HAT) was prepared according to literature procedures.^[1,2]^

**STM/AFM Measurements.** Experiments were carried out with a low-temperature scanning tunneling and atomic force microscope (STM/AFM) equipped with a qPlus tuning fork (resonance frequency *f*_0_ ≈ 29.1 kHz, spring constant k ≈ 1.8 kNm^−1^, quality factor Q ≈ 3 × 10^4^) in ultra-high vacuum (*p* ≈ 2 × 10^−10^ mbar) and at a temperature of 6.1 K. Bias voltages are given as sample bias with respect to the tip. Positive constant-height offsets Δ*z* correspond to a distance increase with respect to the STM/AFM set point above the substrate. After preparing a clean Au(111) surface from cyclic Ne^+^ sputtering at 1 keV and annealing to 720 K, HAT molecules were deposited at a sample temperature below 10 K and annealed to high temperatures on the manipulator. NaCl islands (about 0.2 bilayers) were co-deposited together with the HAT. A small amount of CO was dosed onto the surface for tip functionalization.

**XPS Measurements**. Experiments have been performed at the ALOISA beamline of the synchrotron light source Elettra in Trieste, Italy.^[3]^ Spectra have been acquired at *p* ≈ 1 × 10^–10^ mbar. BEs have been calibrated using the photoelectron lines of the Au(111) substrate. Photoelectron (PE) spectra have been collected using p-polarized light in near normal emission with the surface oriented at a grazing angle of 4.0˚. The electron analyzer (hemispherical, mean radius of 66 mm, acceptance angle FWHM ~ 2˚) is equipped with a two-dimensional Delay-Line detector for fast data acquisition, and was operated at a pass energy E_P_ = 10 eV (resolution 1 % of E_P_) for any photon energy. The overall energy resolution was set to 160, 140 and 115 meV for photoemission measured at photon energies of 515, 400 and 200 eV, respectively. Real time PE acquisition during annealing was performed by PID controlled temperature ramps at constant heating rate of 0.1˚C/s. The HAT and NaCl depositions were performed by homemade boron nitride crucibles, operated at typical temperature of 160-180˚C and 500˚C, respectively. The deposition rate was monitored by quartz microbalances, assuming effective molecular densities $\rho$_HAT_ = 1.2 g/cm^3^ and $\rho$_NaCl_ = 2.16 g/cm^3^. The coverage relative to the Au(111) surface was determined a posteriori by the accurate quantitative analysis of PE spectra described in next sections.

No difference in the HAT assembly can be found between deposition at room temperature and at cryogenic temperatures (10 K).^[4]^

**DFT calculations**

Density Functional Theory calculations have been carried out using the FHI-AIMS code^[5]^ to investigate the electronic properties and the energetic stability of the one-dimensional chain of the HAT molecule and of its complexes on a Au(111) surface. The latter includes the single monomer and dimer formed by the molecule and with Au and Na adatoms, respectively.

We performed the total energy DFT calculations of the (HAT)_2_-Na chain on the Au(111) surface at the GGA-PBE^[6]^ level taking into account the Tkatchenko-Scheffler treatment of the van der Waals interactions.^[7]^ We employed an 8x10 supercell of Au(111) surface, made of three Au layers. All atoms of the supercell structure were optimized, except for the bottom Au layer. The calculations were converged when the remaining atomic forces and the total energy were found below 10^-2^ eV/Å and 10^-5^ eV respectively. We used a single gamma point to sample the Brillouin zone.

Results and discussion

**Table S1**. Hirshfeld and Mulliken charges of Au or Na atom in the isolated states (adatom) and in the associated states ((HAT)_1,2_‑ Au/Na complexes)

|  | Au  (Hirshfeld) | Na  (Hirshfeld) | Au  (Mulliken) | Na  (Mulliken) |
| --- | --- | --- | --- | --- |
| Adatom on Au(111) | 0.01 | 0.39 | -0.05 | 0.50 |
| (HAT)_1_-adatom on Au(111) | 0.10 | 0.31 | 0.03 | 0.54 |
| (HAT)_2_-adatom on Au(111) | 0.15 | 0.23 | 0.12 | 0.57 |
| HAT-(adatom)_3_ on Au(111) | -- | 0.32 | -- | 0.54 |

**Table S2**. Binding energies (*E*_bind_) of HAT monomer and dimer with Ag, Au and Na adatoms on a Ag(111) and Au(111) surfaces.

| *E*_b_ [eV] | Ag, Ag(111) | Au, Au(111) | Na, Ag(111) | Na, Au(111) |
| --- | --- | --- | --- | --- |
| monomer | -2.46 | -2.43 | -2.89 | -2.87 |
| dimer | -4.82 | -4.63 | -5.72 | -5.64 |


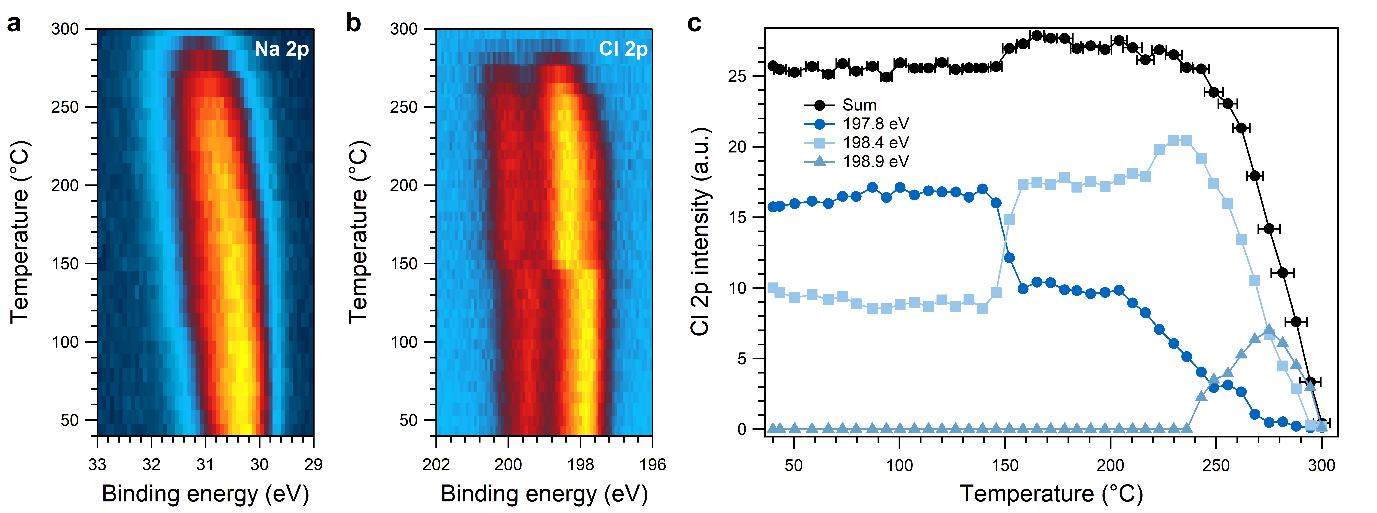


**Figure S1**. Temperature-programmed XPS analysis of 0.3 BL NaCl/Au(111) during annealing from 40 to 300 °C (heating rate: 0.1 °C/s, photon energy: 200 eV ($\Delta$*E* = 115 meV) for Na 2p, 400 eV ($\Delta$*E* = 140 meV) for Cl 2p, acquisition time: 90 s per spectrum): waterfall plots of the Na 2p (a) and the Cl 2p (b) core level spectra as a function of temperature. Colour gradients from dark to light blue indicate low intensities, while high intensities are represented by red to yellow shades. The Cl 2p spectra in panel (b) are deconvoluted into three spin-orbit-split components, each associated with distinct NaCl structural configurations on Au(111).^[8]^ The Cl 2p_3/2_ binding energies are highlighted in (c), where the colour scheme is consistent with Fig. 2 of the main text, which illustrates example fits of the Cl 2p spectra. A significant structural transformation in the NaCl layers occurs at ~150 °C with an increasing coverage of the trilayer NaCl fraction on Au(111) at the cost of bilayer NaCl. Upon further heating above 240 °C, a new component at higher binding energy (198.9 eV) emerges, which is attributed to rock salt formation.^[9]^ At the same temperature, both Na 2p and Cl 2p intensities decrease, signalling the onset of NaCl desorption as neutral molecules. Complete desorption of NaCl is observed by 300 °C, leaving the Au(111) substrate free of NaCl.


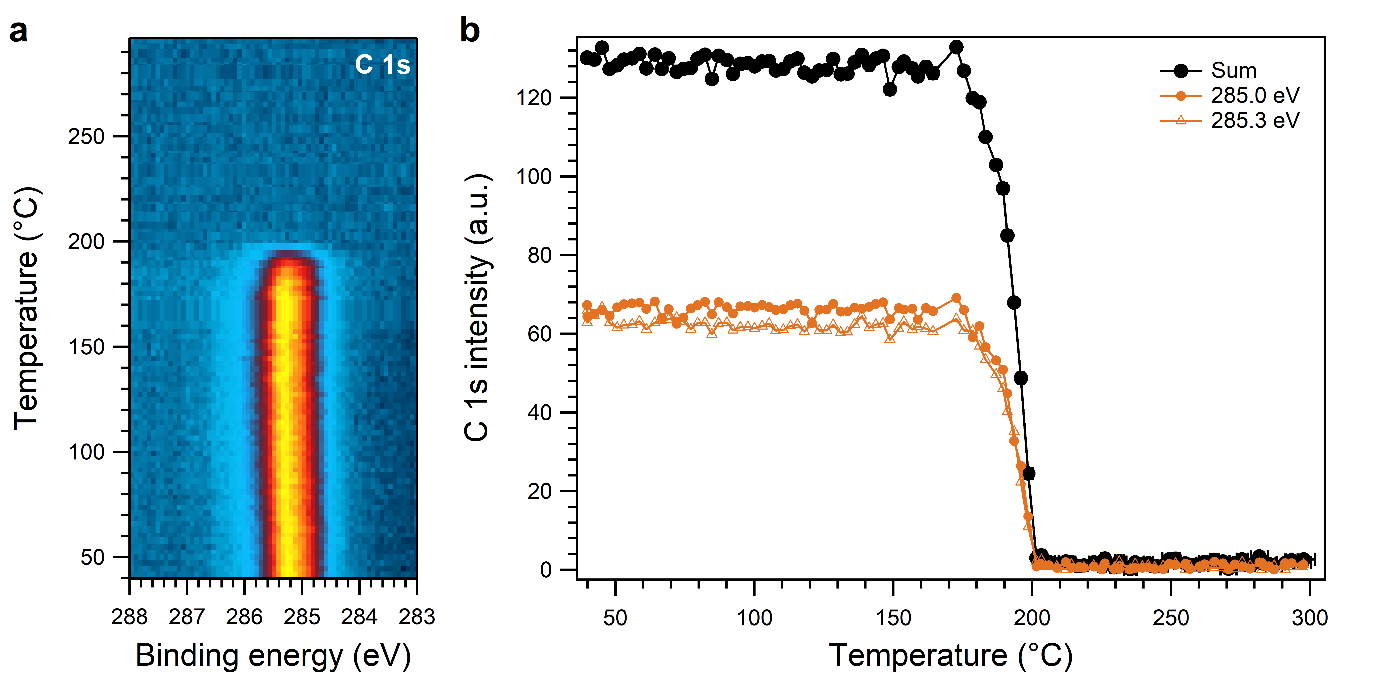


**Figure S2.** Temperature-programmed XPS analysis of 1.2 ML HAT/Au(111) during annealing from 40 to 300 °C (heating rate: 0.1 °C/s, photon energy: 400 eV ($\Delta$*E* = 140 meV), acquisition time: 90 s per spectrum): (a) Waterfall plot of the C 1s core level spectra as a function of the temperature, with colour gradients from dark to light blue representing low intensities and red to yellow indicating high intensities. The C 1s spectra in panel (a) were deconvoluted by two components of equal intensity corresponding to the two chemically distinct carbon atoms in HAT: 6 carbon atoms in the inner benzene ring and 6 in the outer pyrazine rings. Panel (b) shows the extracted intensities of each C 1s component along with the total C 1s intensity as a function of temperature. Although the peak shape remains largely unchanged, the overall C 1s intensity significantly decreases around 175 °C, indicating substantial desorption. By 200 °C, HAT has completely desorbed from the Au(111) surface.


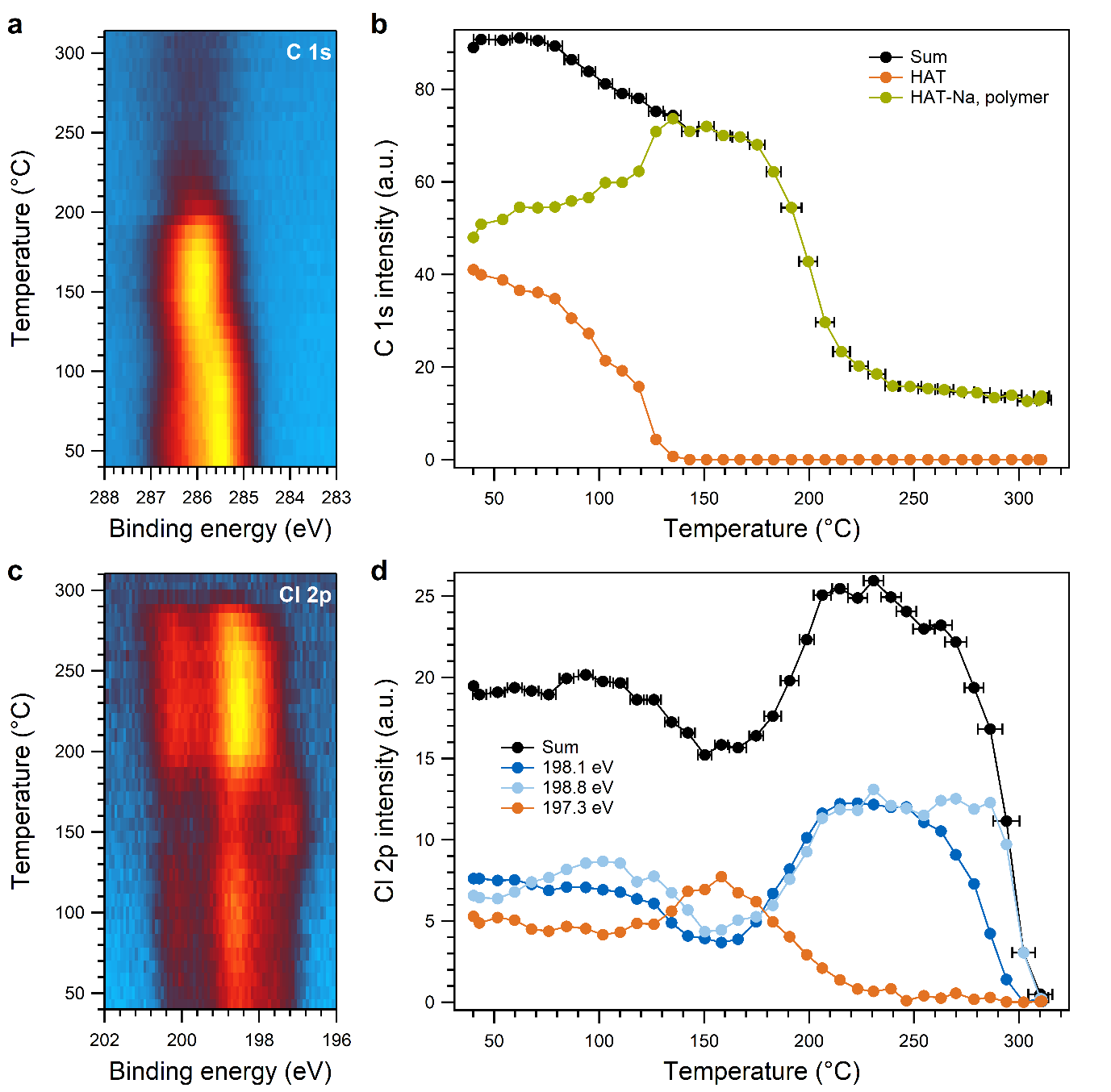


**Figure S3**. Temperature-programmed XPS analysis of 1.2 ML HAT and 0.3 BL NaCl on Au(111) during annealing from 40 to 310 °C (heating rate: 0.1 °C/s, photon energy: 400 eV ($\Delta$*E* = 140 meV), acquisition time: 90 s per spectrum): Waterfall plots of the C 1s (a) and Cl 2p (c) core level spectra as a function of the temperature, with dark to light blue indicating low intensities and red to yellow representing high intensities. The deconvoluted intensities for the C 1s and Cl 2p core levels are plotted in (b) and (d), respectively, with colour codes consistent with the spectral fits in Fig. 2 of the main text. At room temperature, HAT (orange) co-exists with HAT-Na complexes (green), a finding aligned with the presence of free chlorine (orange) alongside NaCl (blue). This coexistence reflects an excess of NaCl deposited on HAT/Au(111) beyond what is required for HAT-Na complex formation. Between 130 and 150 °C, an increasing fraction of free chlorine suggests NaCl dissolution, concurrent with HAT-Na complex formation. As the temperature rises above 170 °C, the decomposition of HAT-Na complexes begins, leading to HAT desorption and the recombination of Na and Cl to form NaCl. NaCl then desorbs at temperatures exceeding 270 °C. By 300 °C, residual HAT polymers and Na remain on the Au(111) surface, while Cl is fully desorbed. The inverse relationship between the total Cl 2p intensity and the intensity of the Cl/Au(111) component (orange) is attributed to chlorine forming a surface chloride layer on Au(111), with part of the chloride located below the surface.^[10]^

The reaction of higher coverages of both HAT (> 1 ML) and NaCl (~ 0.3 BL) was investigated using temperature-programmed XPS experiments, with detailed results provided in Fig. S3. Interestingly, the evolution of the reactant, intermediate, and product species during this process can be tracked by the temperature-dependent changes in the Cl 2p core level, despite the role of a spectator in the HAT polymerization. Free chlorine on Au(111) co-exists with NaCl at room temperature, as the amount of NaCl deposited on HAT/Au(111) exceeds the amount of Na required for the HAT-Na complex formation. Between 130 and 150 °C, the increasing fraction of free chlorine indicates the dissolution of NaCl, concurrent with the ongoing HAT-Na complex formation. In contrast to the situation depicted in Fig. 2 with coverages fairly below 1 ML where NaCl is fully decomposed at room temperature, the high coverages of HAT and NaCl may lead to an activation barrier of the complex formation and NaCl decomposition, consistent with the presence of HAT molecules up to 130 °C. Similar to Fig. 2, a further increase of the temperature above 170 °C initiates the decomposition of HAT-Na complexes accompanied by HAT desorption and recombination of Na and Cl to form NaCl, which desorbs at temperatures above 270 °C. At 300 °C, a fraction of HAT polymers and Na remains on the Au(111) surface, while Cl is entirely desorbed. An intriguing observation is the inverse relationship between the Cl 2p total intensity and the Cl 2p intensity of the component related to Cl/Au(111). This phenomenon is also evident in Fig. 2 where the Cl 2p total intensity increases by a factor of almost two upon the formation of NaCl at the expense of free Cl. As suggested by Gao et al.,^[10]^ Cl adsorbed on Au(111) can form a surface chloride, i.e., part of the chloride is below the surface, increasing the surface area available for HAT-Na complex formation, and thus promoting the HAT polymerization.

The Cl 2p decrease occurring around 150 °C arises from formation of a Cl-rich ad-phase intermixed with the topmost Au layer and attenuation by the 1.2 ML HAT overlayer. Upon annealing above 200 °C, partial HAT desorption exposes more NaCl, leading to recovery and eventual overshoot of the Cl 2p intensity.

At higher coverages, the role of NaCl becomes evident: NaCl decomposes, and HAT-Na complexes form below 150 °C. At higher temperatures, two competing reactions occur: the favored polymerization of HAT and the decomposition of HAT-Na complexes, leading to the desorption of HAT but also to the recombination of NaCl. Thus, by maintaining the temperature below the NaCl desorption threshold of 270 °C, the repeated adsorption of HAT may enable catalytic cycles, thereby enhancing polymer yield.

**Evaluation of XP spectra**

The Na 2p and Cl 2p core level spectra were deconvoluted using Voigt functions, while the C 1s and N 1s core levels were fitted with Doniach-Sunjic functions. A linear background was subtracted from all spectra. The Lorentzian width was fixed between 0.2 and 0.4 eV for each distinct component and core level. The Gaussian width was optimized to best reproduce the peak shapes of the reference samples NaCl/Au(111) and HAT/Au(111), and subsequently adjusted for the complex and polymer samples. In these latter cases, the broader linewidth reflects a distribution of slightly different chemical environments experienced by HAT molecules upon Na complexation.

For the HAT molecule adsorbed on Au(111), the asymmetry of the two C 1s components for the HAT molecule on Au(111) was adjusted to achieve an intensity ratio of 1:1 consistent with the presence of two chemically distinct carbon environments (6 carbon atoms in the inner benzene ring, 6 in the outer pyrazine rings). A similar peak asymmetry was used for the deconvolution of the N 1s core level. In the case of Au 4f, Voigt functions combined with a Shirley background were applied, identifying the bulk component (Au 4f_7/2_ at 84.0 eV) and, in addition, a surface core level peak with an energy shift of 0.35 eV towards lower binding energy in agreement with the literature.^[11]^ Using the obtained peak areas, we outline two approaches to quantify the coverages of the HAT/Au(111) and the NaCl/Au(111). Based on these values, the HAT and NaCl evaporator was calibrated and the coverages of the co-deposited HAT and NaCl on Au(111) are accessible assuming laterally co-existing phases.

**Attenuation of the Au 4f signal**

The coverages of HAT and NaCl of the temperature-programmed XPS (TXPS) experiments were determined based on the attenuation of the Au 4f core level due the adsorbates. The Au 4f intensity was measured both before and after the temperature ramp, using identical beamline and analyser settings. Since complete desorption of the adsorbates was achieved, the Au 4f signal intensity after the TXPS experiment serves as the reference intensity for a clean Au(111) surface. The attenuation of the Au 4f signal corresponding to a full monolayer (ML) of adsorbate A on Au(111) is described by the following expression:

$$\frac{I_{1 ML A/Au(111)}^{Au 4f}}{I_{Au(111)}^{Au 4f}}=\exp\left( -\frac{d_{A}}{\lambda_{A}\left( Au 4f \right)} \right)$$

Here, $I_{Au(111)}^{Au 4f}$ denotes the Au 4f intensity of the clean Au(111) surface, while $I_{1 ML/Au(111)}^{Au 4f}$ represents the intensity attenuated by a full coverage of adsorbate $A$. $d_{A}$ is the thickness of the adsorbate layer which was estimated as $d_{HAT}=2.6 Å$ for HAT on Au, whereas the effective thickness of $d_{NaCl}=5.5 Å$ is used for the NaCl layer, corresponding to bilayer (BL). The latter assumption is used for simplicity, as NaCl has been observed to grow as a mixture of monolayer and bilayer (100)-terminated NaCl films at room temperature.^[12]^ Note that the coverages are defined as the Au(111) surface area fraction which is covered by the adsorbate, i.e., $\theta=1$ amounts to the saturation coverage of HAT and also to the Au(111) surface fully covered by bilayer NaCl.

The signal attenuation lengths of Au 4f photoelectrons in NaCl and HAT were calculated at their respective kinetic energy based on the TPP-2M model.^[13]^ For HAT, parameters for graphite were used. A summary of all signal attenuation lengths used in this analysis is provided in Table S3.

Comparing the theoretical attenuation of the Au 4f signal induced by 1 ML HAT or 1 BL NaCl with the experimentally observed attenuation allows the calculation of the adsorbate coverages $\theta_{A}$ according to the following equation:

$$\theta_{A}=\left( 1-\frac{I_{A/Au(111)}^{Au 4f}}{I_{Au(111)}^{Au 4f}} \right)/ \left( 1-\frac{I_{1 ML A/Au\left( 111 \right)}^{Au 4f}}{I_{Au\left( 111 \right)}^{Au 4f}} \right) (1)$$

Note that this calculation requires the knowledge of the absolute Au 4f photoemission yield from the uncovered Au(111) surface. Since this value was not accessible in every data set of the experiment, an alternative quantification procedure is outlined in the following.

**Quantification of intensity ratios of different core levels**

Relative core level intensities of different species can be used to quantify the elemental composition on the Au(111) surface at each given temperature. The advantage of this procedure is that not only alignment uncertainties but also variations of the photon flux and detection efficiency cancel out. Following this approach, the intensity ratio of the core level $X$ of an adsorbate $A$ and the Au 4f core level ($\frac{I_{A}}{I_{Au 4f}}$) can be expressed as follows:

$$\frac{I_{X}}{I_{Au 4f}}=\frac{N_{A}}{N_{Au}}\cdot\frac{\sigma_{X}}{\sigma_{Au 4f}}\cdot\frac{L_{X}\left( \gamma\right)}{L_{Au 4f}\left( \gamma\right)}\cdot\frac{f\left( \lambda\left( X \right) \right)}{f\left( \lambda\left( Au 4f \right) \right)}$$

The intensity ratio depends on the number of atoms $A$, $N_{A}$, the photoionization cross-section $\sigma_{X}$ of the core level $X$, the angular asymmetry factor $L_{X}(\gamma)$ and a function $f\left( \lambda\left( X \right) \right)$ that accounts for sample geometry and photoelectron attenuation. Relevant parameters are summarized in Table S3 and S4.

The angular asymmetry factor $L_{x}(\gamma)$ is dependent on the angle between the polarization direction of the synchrotron light beam and the direction of photoelectron emission $\gamma$ which amounts 0° in the present experiment:^[14]^

$$L_{x}\left( \gamma=0^{\circ} \right)=1+\beta_{x}$$

The atomic subshell asymmetry parameter $\beta_{x}$ is taken from Yeh and Lindau.^[15]^

For HAT, the function $f\left( \lambda\left( C 1s \right) \right)$ equals one as adsorbed HAT resembles the outermost layer, leaving C 1s photoelectrons unattenuated.

The functions $f\left( \lambda\left( Cl 2p \right) \right)$ and $f\left( \lambda\left( Na 2p \right) \right)$ for a NaCl bilayer on Au(111) are expressed as a sum of two terms. In the first layer, function equals one as the photoelectrons from Cl and Na experience no attenuation. Photoemission from the second layer, however, is damped by the top layer in dependence on the signal attenuation length of Cl 2p or Na 2p photoelectrons in NaCl and the distance between the first and second NaCl layer which is approximated by half a NaCl bilayer thickness $\frac{d_{NaCl}}{2}$:

$$f\left( \lambda_{NaCl}\left( Cl 2p/Na 2p \right) \right)=1+\exp\left( -\frac{d_{NaCl\left( 100 \right)}}{2\cdot\lambda_{NaCl}\left( Cl 2p/Na 2p \right)} \right)$$

For a clean Au(111) surface, $f\left( \lambda\left( Au 4f \right) \right)$ can be written as a sum over all $n$ Au(111) layers below the surface, each attenuated exponentially by the signal attenuation length of Au 4f photoelectrons in Au:

$$f_{clean}\left( \lambda\left( Au 4f \right) \right)=\sum_{n=0}^{\infty} \exp\left( -\frac{n\cdot d_{Au(111)}}{\lambda_{Au}\left( Au 4f \right)} \right)=\frac{1}{1-\exp\left( -\frac{d_{Au(111)}}{\lambda_{Au}\left( Au 4f \right)} \right)}$$

where the distance between adjacent Au(111) layers $d_{Au(111)}$ is 2.35 Å.^[16]^

For the Au(111) surface fully covered by an adsorbate $f\left( \lambda\left( Au 4f \right) \right)$ has to be replaced by:

$$f\left( \lambda\left( Au 4f \right) \right)=f_{clean}\left( \lambda\left( Au 4f \right) \right)\cdot\exp\left( -\frac{d_{A}}{\lambda_{A}\left( Au 4f \right)} \right)$$

Based on the intensity ratios of the Cl 2p, Na 2p, C 1s and the Au 4f photoemission and utilizing the photoionization cross-sections, angular asymmetry factors and photoelectron attenuation functions, the atomic ratios $\frac{N_{Cl}}{N_{Au}}$, $\frac{N_{Na}}{N_{Au}}$ and $\frac{N_{C}}{N_{Au}}$ can be determined. These ratios are then converted to calculate the coverages of NaCl and HAT, $\theta_{NaCl}$ and $\theta_{HAT}$, respectively.

The conversion of the Cl/Au or Na/Au atomic ratio into a NaCl coverage $\theta_{NaCl}$ is based on the unit cell sizes of Au(111) and NaCl(100) which are 7.2 Å^2^ ($A_{Au\left( 111 \right)}$) and 27.0 Å^2^ ($A_{NaCl\left( 100 \right)}$), respectively. Note that the NaCl unit cell contains two Na and two Cl atoms, whereas the Au unit cell hosts one Au atom:

$$\theta_{NaCl}=\frac{N_{Cl/Na}}{N_{Au}}\cdot\frac{A_{NaCl\left( 100 \right)}}{2\cdot A_{Au\left( 111 \right)}}$$

The HAT coverage $\theta_{HAT}$ is calculated using an estimated area of 80 Å^2^ per HAT molecule on Au(111) (based on the AFM image in the inset of Figure 1a) and a stoichiometry of 12 carbon atoms per HAT molecule:

$$\theta_{HAT}=\frac{N_{C}}{N_{Au}}\cdot\frac{A_{HAT}}{12\cdot A_{Au\left( 111 \right)}}$$

So far, the Au 4f intensity of the bare Au(111) surface has been used. However, this value is directly accessible only in TXPS experiments. In such cases, the initial photoemission intensity originating from an adsorbate core level can be related to the Au 4f intensity after desorption.

For other experiments, the Au 4f intensity of the clean surface is typically not acquired under the same conditions as the covered surface so that the intensity originating from the adsorbate can only be related to the Au 4f intensity of the same data set, i.e., the Au 4f photoemission which is attenuated by the covering adsorbate. To account for the attenuation, an iterative approach is employed: First, the coverage is calculated using the measured Au 4f intensity and the attenuation function of the clean Au(111) surface $f_{clean}\left( \lambda\left( Au 4f \right) \right)$. This coverage estimate is then used to calculate the ratio of the Au 4f intensity beneath the adsorbate to that of the clean surface $\frac{I_{A/Au(111)}^{Au 4f}}{I_{Au(111)}^{Au 4f}}$ based on equation (1). The updated ratio is then used to recalculate an improved adsorbate coverage. This iterative process continues until convergence.

Notably, the adsorbate coverages derived from Au 4f attenuation, intensity ratios of the adsorbate and Au 4f core level of the clean surface, and the iterative procedure yield reproducible values with deviations of less than 10%.

**Table S3:** Signal attenuation lengths $\lambda_{A}(X)$ of photoelectrons emitted from the core level $X$ in the material $A$. The signal attenuation length is dependent on the kinetic energy of the photoelectrons $E_{kin}(X)$, which is related to the photon energy $h\nu$. For the signal attenuation length in Au, values provided by Jablonski et al. were used.^[17]^ All other parameters were calculated based on the TPP-2M model.^[13]^

| Signal attenuation length | $h\nu$ (eV) | $E_{kin}(X)$ (eV) | $\lambda_{A}(X)$ (Å) |
| --- | --- | --- | --- |
| $\lambda_{NaCl}\left( Au 4f \right)$ | 200 | 116 | 7.67 |
| $\lambda_{NaCl}\left( Au 4f \right)$ | 400 | 316 | 13.6 |
| $\lambda_{Au}\left( Au 4f \right)$ | 200 | 116 | 4.66 |
| $\lambda_{Au}\left( Au 4f \right)$ | 400 | 316 | 6.28 |
| $\lambda_{NaCl}\left( Cl 2p \right)$ | 400 | 200 | 10.2 |
| $\lambda_{NaCl}\left( Na 2p \right)$ | 200 | 170 | 9.28 |
| $\lambda_{HAT}\left( Au 4f \right)$ | 400 | 316 | 11.6 |

**Table S4.** Photoionization cross-sections $\sigma_{X}$ and atomic subshell asymmetry parameters $\beta_{X}$ of the core levels $X$ at the given photon energy $h\nu$. The parameters are taken from Yeh and Lindau.^[15]^

| Core level | $h\nu$ (eV) | $\sigma_{X}$ (Mb) | $\beta_{X}$ |
| --- | --- | --- | --- |
| Au 4f | 200 | 2.274 | 0.51 |
| Au 4f | 400 | 4.75 | 0.45 |
| Na 2p | 200 | 1.074 | 1.49 |
| Cl 2p | 400 | 1.228 | 1.44 |
| C 1s | 400 | 0.464 | 2 |

**Table S5.** C 1s binding energy and gaussian linewidth for the spectra shown in Fig. 2.

|  | **Binding energy (eV)** | | | | **Gaussian width (eV)** | | | |
| --- | --- | --- | --- | --- | --- | --- | --- | --- |
| spectrum | HAT I | HAT II | complex | polymer | HAT I | HAT II | complex | polymer |
| Au(111) | - | - | - | - | - | - | - | - |
| + HAT | 284.92 | 285.25 | - | - | 0.371 | 0.269 | - | - |
| + NaCl | 284.92 | 285.25 | 285.86 | - | 0.371 | 0.269 | 0.974 | - |
| 130 °C | 284.92 | 285.25 | 285.86 | - | 0.371 | 0.269 | 0.948 | - |
| 180 °C | 284.92 | 285.25 | 285.79 | - | 0.371 | 0.269 | 1.007 | - |
| 250 °C | - | - | - | 286.29 | - | - | - | 1.641 |
| 300 °C | - | - | - | 286.23 | - | - | - | 1.938 |

**Table S6.** N 1s binding energy and gaussian linewidth for the spectra shown in Fig. 2.

|  | **Binding energy (eV)** | | | **Gaussian width (eV)** | | |
| --- | --- | --- | --- | --- | --- | --- |
| spectrum | HAT | complex | polymer | HAT | complex | polymer |
| Au(111) | - | - | - | - | - | - |
| + HAT | 398.76 | - | - | 0.346 | - | - |
| + NaCl | 398.82 | 399.76 | - | 0.346 | 0.842 | - |
| 130 °C | 398.82 | 399.7 | - | 0.346 | 0.944 | - |
| 180 °C | 398.82 | 399.59 | - | 0.346 | 1.013 | - |
| 250 °C | - | - | 400.14 | - | - | 1.923 |
| 300 °C | - | - | 400.17 | - | - | 1.894 |

**Table S7.** Na 2p binding energy and gaussian linewidth for the spectra shown in Fig. 2.

|  | **Binding energy (eV)** | | | **Gaussian width (eV)** | | |
| --- | --- | --- | --- | --- | --- | --- |
| spectrum | complex | NaCl | polymer | complex | NaCl | polymer |
| + NaCl | 30.21 | - | - | 0.662 | - | - |
| 180 °C | 30.21 | 30.86 | - | 0.662 | 0.844 | - |
| 250 °C | 30.21 | 30.79 | - | 0.662 | 1.079 | - |
| 300 °C | - | - | 30.93 | - | - | 0.94 |

**Table S8.** Cl 2p binding energy and gaussian linewidth for the spectra shown in Fig. 2.

|  | **Binding energy (eV)** | | | | | |
| --- | --- | --- | --- | --- | --- | --- |
| spectrum | NaCl bilayer  Cl 2p_3/2_ | NaCl bilayer  Cl 2p_1/2_ | NaCl multilayer  Cl 2p_3/2_ | NaCl multilayer Cl 2p_1/2_ | Cl ad.  Cl 2p_3/2_ | Cl ad. 2p_1/2_ |
| Au(111) | - | - | - | - | - | - |
| + NaCl | - | - | - | - | 197.47 | 199.06 |
| 130 °C | - | - | - | - | 197.38 | 198.97 |
| 180 °C | 198.10 | 199.68 | 198.67 | 200.29 | 197.40 | 199.00 |
| 250 °C | 198.10 | 199.68 | 198.67 | 200.29 | 197.40 | 199.00 |
| 300 °C | - | - | - | - | - | - |

|  | **Gaussian width (eV)** | | | | | |
| --- | --- | --- | --- | --- | --- | --- |
| spectrum | NaCl bilayer  Cl 2p_3/2_ | NaCl bilayer  Cl 2p_1/2_ | NaCl multilayer  Cl 2p_3/2_ | NaCl multilayer Cl 2p_1/2_ | Cl ad.  Cl 2p_3/2_ | Cl ad. 2p_1/2_ |
| Au(111) | - | - | - | - | - | - |
| + NaCl | - | - | - | - | 0.787 | 0.777 |
| 130 °C | - | - | - | - | 0.794 | 0.804 |
| 180 °C | 0.70 | 0.75 | 0.75 | 0.673 | 0.659 | 0.650 |
| 250 °C | 0.70 | 0.75 | 0.65 | 0.676 | 0.650 | 0.750 |
| 300 °C | - | - | - | - | - | - |

**References**

[1] Z. Y. Xiao, X. Zhao, X. K. Jiang, Z. T. Li, *Langmuir* **2010**, *26*, 13048–13051.

[2] D. Rogers, *J. Org. Chem.* **1986**, *51*, 3904–3905.

[3] L. Floreano, A. Cossaro, R. Gotter, A. Verdini, G. Bavdek, F. Evangelista, A. Ruocco, A. Morgante, D. Cvetko, *J. Phys. Chem. C* **2008**, *112*, 10794–10802.

[4] L. L. Patera, X. Liu, N. Mosso, S. Decurtins, S.-X. Liu, J. Repp, *Angew. Chem. Int. Ed.* **2017**, *56*, 10786–10790.

[5] V. Blum, R. Gehrke, F. Hanke, P. Havu, V. Havu, X. Ren, K. Reuter, M. Scheffler, *Comput. Phys. Commun.* **2009**, *180*, 2175–2196.

[6] J. P. Perdew, K. Burke, M. Ernzerhof, *Phys. Rev. Lett.* **1996**, *77*, 3865.

[7] A. Tkatchenko, M. Scheffler, *Phys. Rev. Lett.* **2009**, *102*, 073005.

[8] K. Lauwaet, K. Schouteden, E. Janssens, C. Van Haesendonck, P. Lievens, *J. Phys. Condens. Matter* **2012**, *24*, 475507.

[9] K. Kishi, H. Kirimura, Y. Fujimoto, *Surf. Sci.* **1987**, *181*, 586–595.

[10] W. Gao, T. A. Baker, L. Zhou, D. S. Pinnaduwage, E. Kaxiras, C. M. Friend, *J. Am. Chem. Soc.* **2008**, *130*, 3560–3565.

[11] P. Heimann, J. Van der Veen, D. Eastman, *Solid State Commun.* **1981**, *38*, 595–598.

[12] M. Imai-Imada, H. Imada, K. Miwa, J. Jung, T. K. Shimizu, M. Kawai, Y. Kim, *Phys. Rev. B* **2018**, *98*, 201403.

[13] S. Tanuma, C. J. Powell, D. R. Penn, *Surf. Interface Anal.* **1994**, *21*, 165–176.

[14] D. J. Kennedy, S. T. Manson, *Phys. Rev. A* **1972**, *5*, 227.

[15] J. Yeh, I. Lindau, *At. Data Nucl. Data Tables* **1985**, *32*, 1–155.

[16] W. P. Davey, *Phys. Rev.* **1925**, *25*, 753.

[17] C. J. Powell, A. Jablonski, *J. Phys. Chem. Ref. Data* **1999**, *28*, 19–62.
